# Rethinking the Tampa scale of kinesiophobia as a measure of re-injury worries after anterior cruciate ligament injury

**DOI:** 10.1186/s13102-026-01684-y

**Published:** 2026-04-06

**Authors:** Adam Grinberg, Martin Björklund, Charlotte K. Häger

**Affiliations:** 1 Department of Community Medicine and Rehabilitation, Umeå University, Umeå, SE-901 87 Sweden; 2 Department for Occupational Health, Psychology and Sports Sciences, Faculty of Health and Occupational Studies, University of Gävle, Gävle, Sweden; 3 Department of Diagnostics and Intervention, Clinics of Orthopaedics, University Hospital of Umeå, Umeå, Sweden

**Keywords:** Fear of re-injury, TSK, ACL, Anxiety, Psychological readiness, Pain, Psychometric properties

## Abstract

**Background:**

The term kinesiophobia originates in the context of the cognitive fear-avoidance model of pain. The Tampa Scale of Kinesiophobia (TSK) is frequently used to assess this construct, notably among populations for which it was not designed, including athletes with anterior cruciate ligament (ACL) injury, for whom pain is not a major concern. The objective of this study was to determine the suitability of the TSK for evaluating re-injury worries in ACL-injured persons with and without pain, by assessing key psychometric properties.

**Methods:**

Ninety-two individuals post-ACL reconstruction (ACLR) were included and divided into PAIN and NO-PAIN subgroups, based on a 90% cutoff on the pain subscale of the Knee Injury and Osteoarthritis Outcome Score. Correlation analyses were employed to assess the contribution of pain-specific TSK items to the total score. Criterion validity (Cohen’s-kappa) was evaluated between an established TSK_total_ cutoff and classification based on the re-injury fear-specific TSK_Q9_, with an optimal cutoff further explored via receiver operating characteristic (ROC) analysis. The TSK’s internal consistency was tested on subgroup level, using Chronbach’s-α. Finally, in a subset of participants, the TSK’s discriminant validity was assessed through correlation with the ACL Return-to-Sport-after-Injury survey (ACL-RSI) of psychological readiness.

**Results:**

Pain-specific TSK items correlated strongly with TSK_total_ (r_s_=0.85). Classification based on a previously recommended TSK_total_ cutoff (38-point) demonstrated fair agreement with TSK_Q9_ (K = 0.31), with low sensitivity and high specificity. An optimal cutoff of 33.5 for TSK_total_ had a sensitivity of 70.6% and specificity of 87.8%. The TSK’s internal consistency was poor (*α* = 0.65) for NO-PAIN and acceptable (*α* = 0.77) for the PAIN subgroup. The TSK_total_ and ACL-RSI scores were not correlated.

**Conclusion:**

The TSK may have limited suitability for individuals after ACLR due to poor internal consistency when pain is not a concern and limited relationship to re-injury fear, regardless of selected cutoff, and psychological readiness. The construct of kinesiophobia is likely less relevant in this population while other more suitable constructs could provide more meaningful assessment of psychological aspects affecting athletic recovery. Clinicians should consider prioritising more efficient, population-specific tools for detecting re-injury worries, over commonly-used but less-fitting tools like the TSK.

**Supplementary Information:**

The online version contains supplementary material available at 10.1186/s13102-026-01684-y.

## Introduction

Kinesiophobia (from Greek: Kinesis-phobos; literally fear of movement) was defined by Kori, Miller and Todd in 1990 as “an excessive, irrational, and debilitating fear of physical movement and activity resulting from a feeling of vulnerability due to painful injury or re-injury” [1]. This psychological construct has since been widely addressed in the context of the cognitive fear avoidance model of pain [2], a framework adopted by researchers and clinicians over the last four decades. Briefly, the model outlines two potential trajectories patients might take when experiencing a painful injury or musculoskeletal condition. In an optimal scenario, the patient confronts their pain and accepts physical activity as a successful pathway to recovery. In the more sinister trajectory, the cognitive and emotional components predominantly exacerbate the patient’s pain experience [2]. They may develop catastrophising thoughts, possibly leading to pain anxiety, avoidance behaviour and to disability and depression [3]. The Tampa Scale of Kinesiophobia (TSK), originally with 17 items (TSK-17) [4] has been used since 1991 as the predominant tool for assessing kinesiophobia among patients with pain-dominating conditions, including chronic low back pain [5], total knee arthroplasty [6], patellofemoral pain [7] and fibromyalgia [8].

Sports researchers and clinical practitioners have however extended the use of kinesiophobia measures to populations for which pain is not a primary concern. Specifically, athletes who have suffered anterior cruciate ligament (ACL) injury are commonly administered the TSK to evaluate fear of re-injury [9]. Re-injury worries (anxiety and fear) are indeed frequent after ACL injury and considered the main reason for not returning to pre-injury activity level [10, 11]. The psychological response to an athletic injury may also subconsciously influence movement patterns [12, 13] and be associated with secondary injuries [14, 15]. The concept of kinesiophobia thus seems appropriate in this context given the risk of potentially hazardous movements as the trigger for a secondary injury. However, fear of re-injury and kinesiophobia are not identical as the latter involves pain as the fear-relevant threat associated with movements. This distinction is critical because in the TSK more than 50% of the items explicitly refer to pain as the major deterrent [4]. The population for which the TSK was developed, patients suffering from musculoskeletal chronic pain, is inherently different from sportspeople recovering from an injury, who generally aspire to resume physical activity or preferably return to their respective sport. Re-injury anxiety among ACL-injured persons is complex, involving many emotions related to a life-changing experience [16, 17], and not limited to worries about the acute injury pain [17–19].

Kvist et al. were the first who administered the TSK to ACL-injured individuals [20]. Notably, they adapted the instrument to the target population, with the word ‘pain’ replaced by ‘knee trouble’ in most instances, among other relevant modifications. The use of the unmodified TSK for the ACL-injured athletic population has however since then become increasingly common, with the number of published papers rising from one in 2005 to a total of 111 in 2025. Furthermore, while the TSK includes several items reflecting different sub-constructs [21] the overall score is predominantly used to quantify an athlete’s worries of re-injury [9].

A recent study [12] employed a reductionistic approach to assessing re-injury fear by stratifying individuals following ACL reconstruction (ACLR) into high or low fear subgroups based on a single item from the TSK-17. The ninth statement from the TSK – “*I am afraid that I might injure myself accidentally*” (TSK_Q9_) – was selected as it explicitly refers to re-injury worries. Individuals classified as “High-fear” exhibited greater muscle co-activation, suggesting a protective strategy during side-hops which challenge knee stability [12]. A classification based on TSK_Q9_, albeit simplistic, may therefore provide a crude estimate of a person’s worries about being re-injuried.

Recognising that the TSK may not fully align with the psychological profile of ACL-injured athletes, the overall purpose of the present study was to empirically evaluate the suitability of the TSK for evaluating re-injury worries in this population. The five specific objectives were to: (1) determine the extent to which the TSK pain-specific items contribute to the total score, whereasa strong contribution was hypothesised, indicating that pain-related information heavily influences the score; (2) evaluate whether any existing ACLR pain translates into kinesiophobia, with be a weak relationship between pain and TSK scores was hypothesised, suggesting that the presence of pain does not necessarily correspond to kinesiophobia in this population; (3) evaluate the criterion validity of the TSK by exploring the agreement between a standard TSK total score cutoff for kinesiophobia [5] and re-injury worries, as determined by TSK_Q9_ cutoff [12, 22], and to explore an optimal TSK cutoff that best distinguishes between individuals with high and low worries. A low agreement between the measures was hypothesised considering that the cutoffs were established for two distinct populations; (4) assess the internal consistency of the TSK among individuals following ACLR with and without pain. Given the construct’s strong dependency on presence of pain, a poor internal consistency was hypothesised, particularly in pain-free individuals; and finally, (5) evaluate the discriminant validity of the TSK by comparison with the ACL Return to Sports after Injury survey (ACL-RSI) [23], a population-specific measure of psychological readiness. Given that the TSK and ACL-RSI assess related yet distinct constructs, a weak to no relationghip was anticipated between the measures.

## Methods

This was a cross-sectional study performed at the Department of Community Medicine and Rehabilitation, at Umeå University, Sweden. Participant data were collected between 2015 and 2024, in two separate projects involving series of functional testing for individuals who had suffered a unilateral ACL injury. The present study is a secondary analysis of self-reported outcomes, which were administered prior to any testing. All participants provided written informed consent according to the declaration of Helsinki and the research was approved by the Swedish Ethical Review Authority (Project No. 2015/67 − 31; 2021–03860).

### Sample size justification

An a-priori power calculation was performed for Cronbach’s α testing based on a formula introduced by Bonett., 2002 [24]. For testing of a 17-item questionnaire, with the null hypothesis of internal consistency comparable to that for chronic pain patients [25] and an alternate hypothesis of a non-satisfactory α of 0.65 (i.e., effect size of 0.2), we estimated a required sample size of 37 participants to achieve 80% power [24]. For construct validity, we followed a recommendation of 50 participants as the minimal sample size [26].

### Participants and subgrouping

**Fig. 1:**
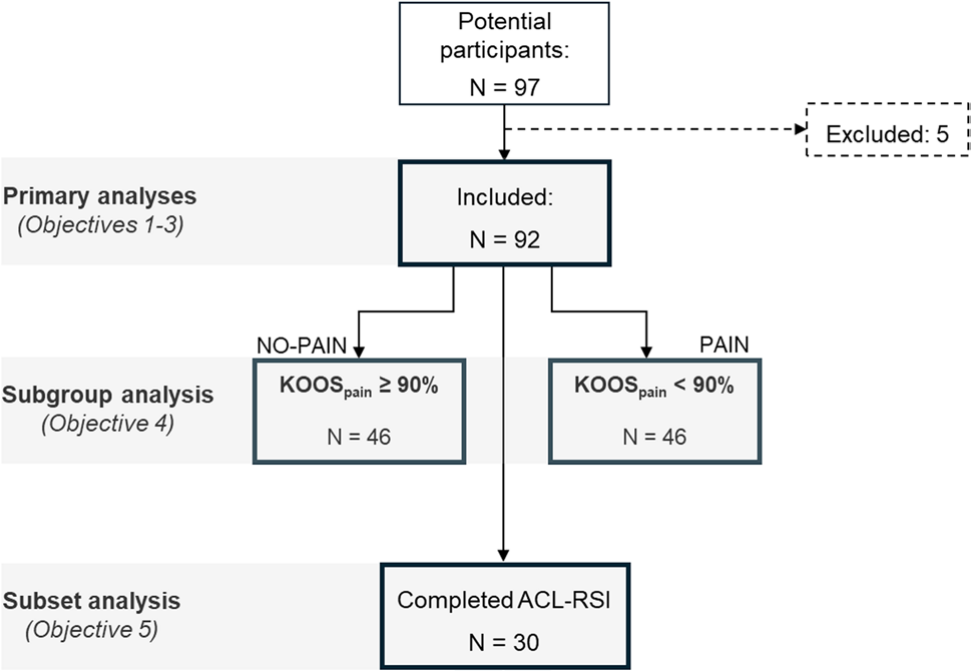
Flowchart of the study design. *Abbreviations*: KOOS, Knee Injury and Osteoarthritis Outcome Score; ACL-RSI, Anterior Cruciate Ligament Return to Sport after Injury Survey

All participants for the two primary projects were recruited using a convenience sampling approach. Individuals who had suffered a unilateral ACL injury were recruited from an orthopaedic clinic at the regional hospital, from a private sports medicine clinic, via ads spread around the university campus, social media platforms and word-of-mouth. Inclusion criteria included: 15–35 years of age who suffered a unilateral ACL injury treated with reconstructive surgery with hamstring autograft, which is the most common practice in Sweden [27]. For the present study, only participants whose injury occurred during sport participation (either recreational or competitive, during training or competition) were included. Participants were excluded if one or more of their questionnaire responses had irregularities (e.g., missing item, marking a midpoint between two response alternatives or marking two instead of one alternative). Out of 97 potential participants, one individual was excluded as his injury occurred in non-sport-related circumstances and four more were excluded due to irregularities in their forms. Specifically, one participant had eight missing items due to a skipped questionnaire page, another had three instances of double marking, a third marked a midpoint between response alternatives and a fourth participant misunderstood the questionnaires, rendering their data unreliable. A final sample of 92 participants was thus used for the analysis (Fig. 1). Subgroup allocation was then performed based on the Pain subscale of the KOOS_pain_. The KOOS is commonly used to evaluate both short and long-term knee injury-associated outcomes [28], and was considered a relevant patient-reported outcome measure for individuals in the non-acute stages after ACL injury [29]. It consists of five subscales: Symptoms, Pain, Activities of Daily Living, Sports and Recreation function, and Quality of Life. The Pain subscale is comprised of nine items, one referring to general knee pain and eight that refer to functional pain during the last week (i.e., each refers to a different function). Scoring is summed into percentage, with 100% indicating absence of pain. A previous study investigating the development of post-injury osteoarthritis [30] used a KOOS_pain_ threshold of 86.1% to define symptomatic knees. For the present study, a more conservative cutoff of 90% was selected to account for minor residual injury/surgery pain, while providing a stringent basis for subgroup comparisons, particularly given a young active population in the present study. Participants who scored 90–100% were allocated to the NO-PAIN subgroup (*N* = 46) and participants who scored 0–90% were allocated to the PAIN subgroup (*N* = 46). Furthermore, a subset analysis of 30 participants (i.e., only from one of the two projects) was conducted to determine the discriminant validity of the TSK – the degree to which it diverges from another measure of a different construct. For this purpose, we tested the relationship between the TSK and the ACL-RSI questionnaire [23], a condition-specific measure for psychological readiness to return to sports after ACL injury that includes 12 items covering injury-relevant psychological aspects including fear, confidence and nervousness regarding their rehabilitation and returning to their respective sports. This was an exploratory analysis given that the subset completing the ACL-RSI (*n* = 30) was below the recommended minimum of 50 participants [26]. Participant characteristics, including pre-injury and current activity levels (Tegner scores [31]) and time since surgery, are presented in Table 1.

**Table 1:** Participants’ background characteristics with groups (PAIN vs. NO PAIN) determined based on a 90% cutoff on the Knee Injury and Osteoarthritis Outcome Score, Pain subscale. Values are presented as median (interquartile range), unless otherwise stated

	Total (*n* = 92)	NO-PAIN (*n* = 46)	PAIN (*n* = 46)	*P *(Subgroup)	Subset (*n* = 30)	*P *(Subset)
Sex (M/F)	31/61	15/31	16/30	NS	5/25	NS
Age (yrs); mean (SD)	24.9 (4.7)	24.2 (4.7)	25.6 (4.6)	NS	24.0 (4.9)	NS
Time post-surgery (months)	13.5 (14.2)	16.4 (15.7)	12.3 (10.8)	0.013	18.1 (12.2)	NS
Pre-injury activity (Tegner, 1–10)	8 (2)	9 (2)	8 (2)	NS	9 (3)	0.007
Current activity (Tegner, 1–10)	6 (3)	6.5 (3)	6 (4)	NS	7 (4.3)	NS

*NS* Non-Significant

### Tampa scale of kinesiophobia (TSK)-derived outcomes

All questionnaires across both projects were identically worded and organised across projects and administered before any physical test occurred. However, the order of administration and data collection delivery formats differed slightly. The Swedish version of the TSK-17 was administered to all participants, either in paper form or using an electronic data capturing tool (REDCap) [32]. Based on participants’ data and after questions 4, 8, 12 and 16 had been given reverse scoring [4], several variables were extracted: 1*)* TSK total score, (TSK_total_) reflecting the general construct of Kinesiophobia. (2) TSK_pain_, a subscore comprised of the ten items (Q2, Q4, Q7, Q8, Q10, Q11, Q12, Q13, Q16, Q17) in which pain is explicitly referred to. (3) Item 9 from the TSK-17 (TSK_Q9_) which specifically addresses being afraid of a future injury [12, 22].

### Statistical analysis

All statistical tests were performed with the Statistical Package for the Social Sciences software (version 28.0, IBM SPSS statistics, Armonk, New York, USA). All questionnaire data, being ordinal in nature, were analysed using non-parametric statistics to provide appropriate and accurate estimates without assuming interval scaling or normal distribution. Descriptive statistics were computed for demographics and main outcome variables for both the entire cohort and per subgroup (NO-PAIN / PAIN; Tables 1 and 2), followed by Mann-Whitney U tests to detect subgroup differences and ηp^2^ for effect sizes. Guided by the five objectives, statistical analyses were performed either on the entire cohort *(primary analyses; objectives 1–3)*, subgroup analysis *(objective 4)* and subset analysis, performed on a subset of 30 individuals *(objective 5)*. *In the primary analyses*, Spearman correlations were calculated between TSK_total_ and TSK_pain_ *(objective 1)* and between TSK_total_ and KOOS_pain_ scores *(objective 2)*. Correlation coefficients were interpreted as negligible (0–0.3), weak (0.3–0.5), moderate (0.5–0.7), strong (0.7–0.9) or very strong (0.9–1) [33]. To account for multiple correlations, Bonferroni corrections were applied to all p-values. For the analysis of criterion validity, the extent to which the TSK agrees with an external criterion of a phenomenon [34], the agreement between TSK_total_ and TSK_Q9_ dichotomisations was tested *(objective 3)*. The TSK_total_ cutoff for kinesiophobia was set at ≥ 38 based on Vlaeyen et al., 1995 [5]. It was then tested for agreement against the TSK_Q9_ cutoff (response alternatives 1–2 = No fear; 3–4 = fear), which has previously been established [12, 22]. Pearson Chi-square test was applied on both grouping assignments to assess them for association, with Cramer V test for measurement of association effect size. The 95% confidence interval (CI) for Cramer’s V was estimated using a non-parametric bootstrap procedure with 5,000 resamples. Effect sizes were interpreted as negligible (0.1 to 0.3), medium (0.3 to 0.5) and large (> 0.5) [35]. Additionally, Cohen’s Kappa was calculated to determine the level of agreement between the two classification methods, and interpreted as poor (0–0.2), fair (0.21–0.4), moderate (0.41–0.6), substantial (0.61–0.8) and strong (0.81–1) [36]. TSK_total_ sensitivity (i.e., number of true-positives – based on TSK_Q9_ classification – divided by all positives) and specificity (i.e., number of true-negatives divided by all negatives) were correspondingly calculated. To further assess the criterion validity of the TSK *(objective 3)*, a receiver operating characteristic (ROC) analysis was employed. The diagnostic performance was evaluated using the area under the curve (AUC), with values interpreted as poor (0.6 ≤ AUC < 0.7), fair (0.7 ≤ AUC < 0.8), considerable (0.8 ≤ AUC < 0.9) or excellent (AUC ≥ 0.9) [37]. An optimal TSK_total_ cutoff was determined according on the maximal Youden J statistic [37]. *For the subgroup analysis*, based on the 90% cutoff in the KOOS_pain_ subscale, the TSK’s internal consistency was assessed using Cronbach’s α separately for each subgroup *(objective 4)*. To confirm that the findings were not dependent on this threshold, a supplementary analysis was performed (*supplementary material*) using the previously published 86.1% cutoff [30]. Values considered acceptable for internal consistency ranged from 0.70 to 0.90 [38]. *For the subset analysis*, between the TSK and the ACL-RSI questionnaire, Spearman correlation was calculated between the two scores *(objective 5)*. For all the comparisons, statistical significance was set to *p* < 0.05.

## Results

### Descriptives

**Fig. 2:**
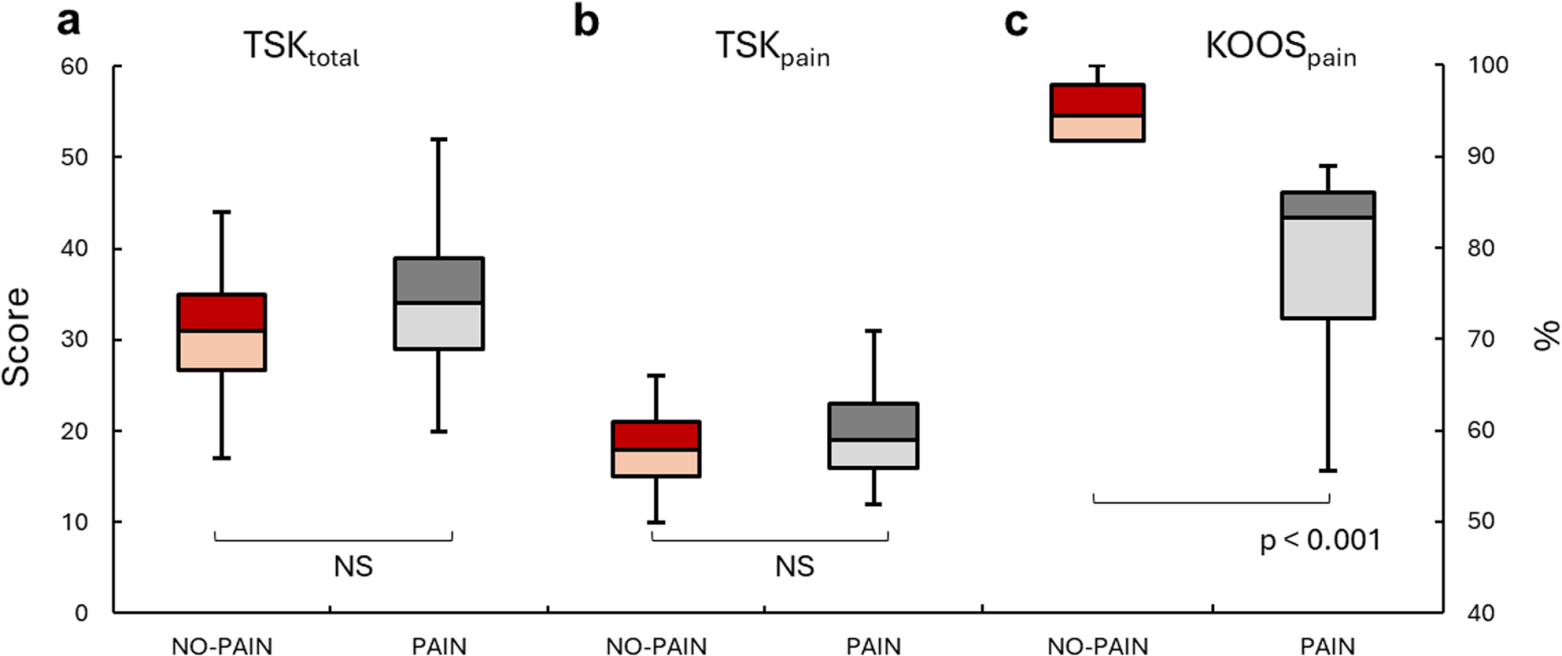
Subgroup comparisons of kinesiophobia and pain-related measures. Boxplots illustrate the distribution of three key scores based on NO-PAIN/PAIN subgroups. The boxes represent interquartile range with median values indicated by horizontal lines. Minimum/maximum values indicated by the whiskers. **a** Tampa scale of kinesiophobia (TSK) total score (TSK_total_; Score range: 17–68, with higher score representing greater kinesiophobia); **b** Summation of the 10 pain-specific items from the TSK (Score range: 17–40, with higher score representing greater kinesiophobia); **c** The Pain subscale of the Knee Injury and Osteoarthritis Outcome Score (KOOS_pain_; used to define the subgroups; lower percentage representing more pain)

There were significant differences between NO-PAIN and PAIN subgroups in all KOOS subscales (*p* < 0.001, ηp^2^ ≥ 0.16), with the PAIN subgroup demonstrating worse overall scores. No subgroup differences were detected in any of the TSK-derived outcomes (Table 2; Fig. 2). The subset of 30 participants used for the discriminant validity analysis was comparable to the rest of the cohort with respect to demographic and clinical characteristics (*p* ≥ 0.080), with only the pre-injury Tegner scorefound to be slightly higher in the subset (median [min-max]: 9 [7–10]) compared to the rest of the participants (8 [4–10]; *P* = 0.007).

**Table 2:** Participants’ questionnaire data and group comparisons (PAIN vs. NO-PAIN). Values are presented as median (interquartile range). Effect sizes are presented for significant results

	Total (*n* = 92)	NO-PAIN (*n* = 46)	PAIN (*n* = 46)	(Subgroup)	Effect size (ηp^2^)	Subset (*n* = 30)	*p* (Subset)
Tampa Scale of Kinesiophobia							
Total (17–68)	33 (9)	31 (8.3)	34 (10)	NS		33.5 (7.8)	NS
Pain (10–40)	18.5 (6)	18 (6)	19 (7)	NS		18.5 (5.3)	NS
Q9 (1–4)	3 (1)	2.5 (1)	3 (2)	NS		3 (2)	NS
Knee Injury and Osteoarthritis Outcome Score (KOOS, 0-100%)							
Symptoms	78.6 (17.9)	85.7 (17.8)	71.4 (20.4)	< 0.001	0.25	78.8 (17.0)	NS
Pain	90.3 (11.1)	94.4 (6.2)	83.3 (13.9)	< 0.001	0.76	91.7 (12.5)	NS
Activities of Daily Living	100.0 (5.2)	100.0 (0.0)	97.1 (8.1)	< 0.001	0.35	100.0 (5.9)	NS
Sports/recreation	80.0 (30.0)	87.5 (21.3)	65.0 (35.0)	< 0.001	0.30	80.0 (26.3)	NS
Quality of Life	62.5 (18.8)	68.8 (25.0)	56.3 (25.0)	< 0.001	0.16	62.5 (14.1)	NS
ACL-Return to Sport after Injury survey (0-100)	–			–		44.6 (30.2)	–

Q9, Question 9 (“I am afraid I might injure myself accidentally”)

Subgrouping was determined based on a 90% cutoff on the KOOS Pain subscale

### TSK correlations – Objectives 1, 2

**Fig. 3:**
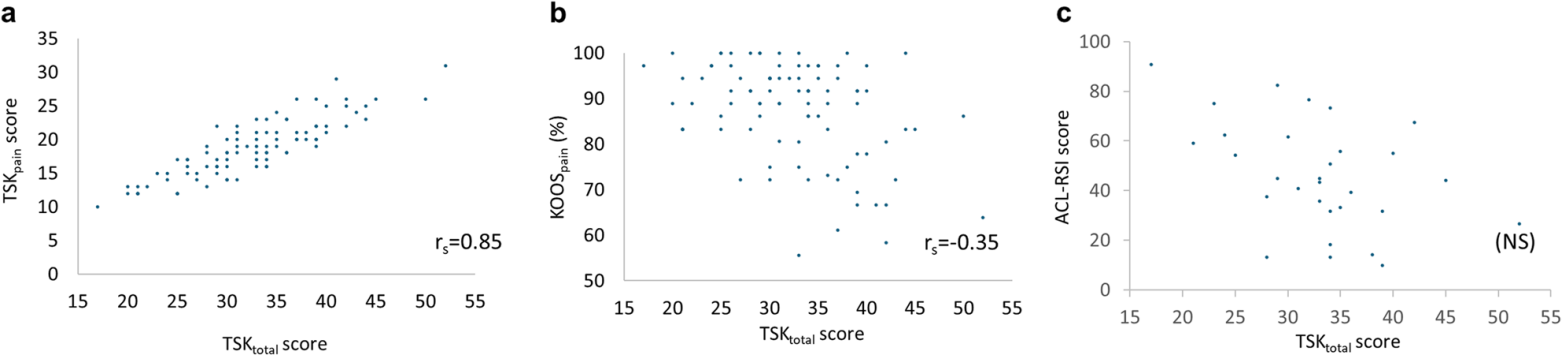
TSK Correlations. **a** Correlation between TSK total score (TSK_total_; Score range: 17–68, with higher score representing greater kinesiophobia) and TSK pain-specific items (TSK_pain_); **b** Correlation between TSK_total_ and self-reported pain level, evaluated using the KOOS pain subscale (TSK_pain_; lower percentage representing more pain) **c** Subset (*N* = 30) analysis – correlation between TSK_total_ and the ACL-RSI measure for psychological readiness. *Abbreviations*: TSK, Tampa Scale of Kinesiophobia; KOOS, Knee Injury an Osteoarthritis Outcome Score; Q9, Question 9; ACL-RSI, Anterior Cruciate Ligament Return to Sport After Injury Survey

TSK_total_ strongly correlated with TSK_pain_ (r_s_ = 0.85, 95% CI: [0.78–0.90], *p* < 0.001; Fig. 3a) while a weak negative correlation was observed between TSK_total_ and KOOS_pain_ (r_s_ = -0.35, 95% CI: [-0.52 – -0.19], *p* = 0.002; Fig. 3b).

### Criterion validity – Objective 3

**Fig. 4:**
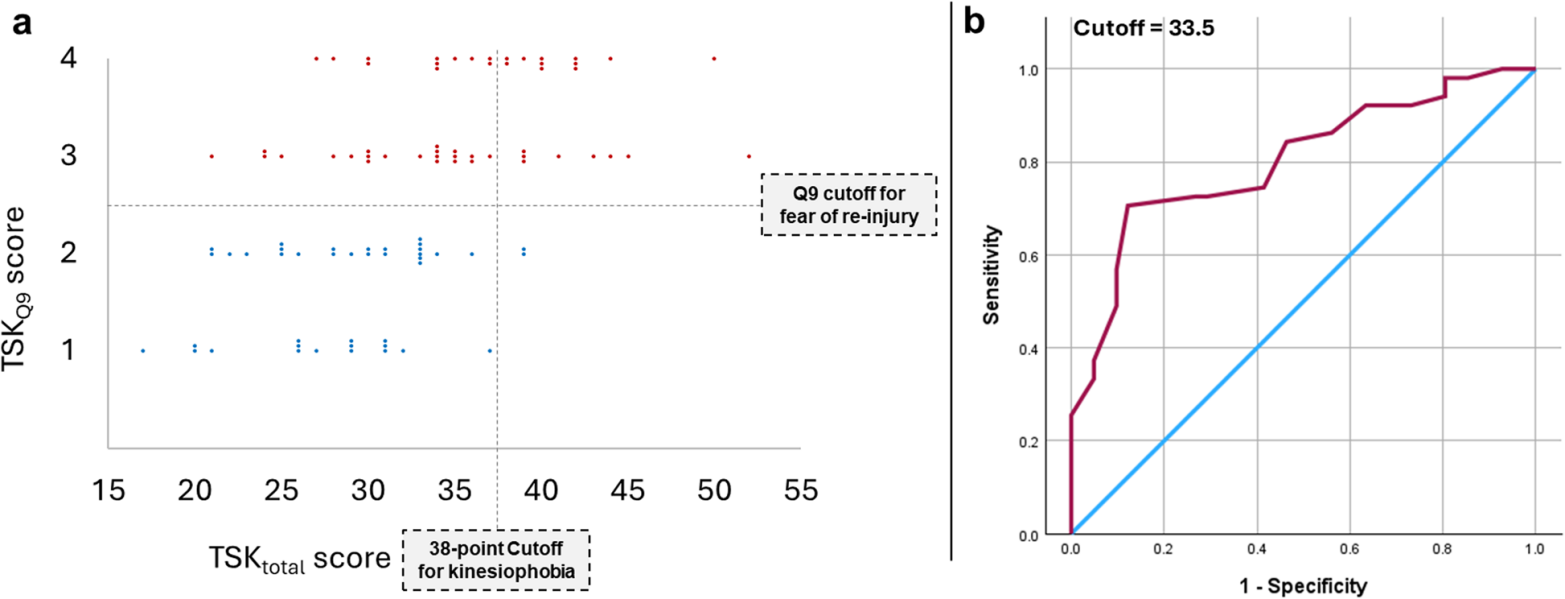
Criterion validity of the TSK. **a** Agreement between binary classifications based on TSK_total_ and TSK_Q9_ is shown, with the TSK_total_ classification (based on a cutoff of 38 for kinesiophobia [5]) demonstrating a sensitivity of 37.25% and specificity of 97.5%. **b** Receiver operating characteristic (ROC) curve representing the classification performance of the TSK-17 total score, against TSK_Q9_-based dichotomisation. The X-axis indicates 1-specificity, (i.e., false positive rate) and the Y-axis indicates sensitivity (true positive rate). The blue diagonal line represents the performance of a random classifier, where the true positive rate equals the false positive rate. The optimal cutoff, the point on the ROC curve with the best sensitivity and specificity (70.6% and 87.8%, respectively), based on the maximum Youden J statistic is indicated. *Abbreviations*: TSK, Tampa Scale of Kinesiophobia; Q9, Question 9

Comparing the TSK_total_ and TSK_Q9_ binary classifications (Fig. 4a) revealed a significant association (χ² = 18.790, *p* < 0.001) between the two measures, though with a medium effect size (Cramer’s V = 0.452, 95% CI: [0.308–0.575], *p* < 0.001). Based on a cutoff score of 38 for kinesiophobia [5], the TSK_total_ had a sensitivity of 37.25% (95% CI: 23.99% − 50.52%) and specificity of 97.50% (95% CI: 88.53% − 100%) against the TSK_Q9_ classification. The Kappa statistic indicated a fair level of agreement between the two classifications. (K = 0.313; 95% CI: [0.172–0.454], *p* < 0.001). ROC analysis on our cohort instead demonstrated a considerable diagnostic performance of the TSK_total_ (AUC = 0.802, 95% CI: 0.712–0.891; Fig. 4b). The maximum Youden (J = 0.584) corresponded to an optimal cutoff of 33.5, yielding sensitivity of 70.6% and specificity of 87.8%.

### Internal Consistency (subgroup analysis) – Objective 4

The TSK Cronbach’s α for the NO-PAIN subgroup (KOOS_pain_ ≥ 90%) was 0.652, indicating a non-satisfactory internal consistency for individuals with minimal or no pain. For the PAIN subgroup, (KOOS_pain_ < 90%), *α* = 0.769 was observed, indicating satisfactory internal consistency.

### Discriminant validity (subset analysis) – Objective 5

For the subset of 30 participants, no significant correlation was observed between TSK_total_ and ACL-RSI scores (Fig. 3d).

## Discussion

The overarching purpose of this study was to establish whether the TSK was a suitable tool to assess re-injury worries among individuals after ACLR, either with or without current pain. The present findings indicate that the pain-specific items of the TSK substantially drive the total score, while at the same time, kinesiophobia and reported pain show a negligible relationship. The TSK had acceptable internal consistency in individuals with pain, but not in those without pain. The TSK’s criterion validity based on an established cutoff-score of 38, had fair agreement with a classification based on TSK_Q9_, while even an optimal cutoff was shown to have a limited capability to detect fearful individuals. Finally, for the subset of 30 participants, the TSK was not associated with the ACL-RSI, reinforcing that the two instruments assess distinct constructs. Collectively, these findings suggest that the TSK may not be the most suitable measure to use with athletic individuals after ACLR, for evaluation of their re-injury worries.

The construct of kinesiophobia relates mainly to other types of musculoskeletal concerns, with its target population being comprised of individuals suffering from pain-dominating conditions [1, 39]. Indeed, the ten items containing an explicit mentioning of pain significantly contributed to the total score. Although half the participants were allocated to a PAIN subgroup based on their KOOS_pain_ scores, the level of their self-reported pain was slightly higher than the cutoff value previously established for symptomatic knees [30]. Moreover, the PAIN subgroup had statistically shorter times from surgery, making the presence of residual surgery pain, as well as lower scores in all other subscales of the KOOS, somewhat expected. This did not influence their kinesiophobia, given similar scores in TSK-derived outcomes, with both groups’ median TSK_total_ scores being below the cutoff for kinesiophobia used for other populations [5]. In addition, the associations between KOOS_pain_ and TSK_total_ scores were low, whereas patients with chronic pain conditions have been shown to have strong associations between kinesiophobia and pain-related outcomes [40]. Together, these findings suggest that subacute pain after ACLR may not translate into anxiety regarding future injuries and instead may be perceived as a natural consequence to the recent trauma. However, given the cross-sectional nature of the present study, no causal relationship can be inferred.

For the NO-PAIN subgroup, the internal consistency of the TSK was low and below the acceptable threshold [38], which raises concerns about its applicability for assessing re-injury worries in this population. Further, although the PAIN subgroup had an acceptable internal consistency, it was considerably lower than α values reported in individuals suffering from chronic pain [25]. This suggests that, particularly for those whose primary concern is not pain, the TSK may not fully capture a single consistent construct and that there may be conceptual differences or lower relevance of certain items.

In previous work [12, 22], which also included individuals after ACLR, the inherent heterogeneity of the TSK items motivated the use of a single question (TSK_Q9_) specifically referring to being afraid of accidently getting injured. With no gold-standard measure for re-injury worries, establishing a cutoff for fear in any questionnaire is problematic. TSK_Q9_ allowed for a simple dichotomisation of participants’ worries, and indeed those classified as “high-fear” demonstrated distinct biomechanics when performing an injury-relevant functional hop task [12, 22]. The present findings indicate that using the TSK_total_ score as a classifier for kinesiophobia among individuals after ACLR is likely going to fail in identifying individuals with re-injury worries, at least with the use of the previously established cutoff [5]. The ROC analysis determined a more sensitive cutoff of 33.5, although with a sensitivity of 70.6% a substantial number of fearful patients would remain unidentified. This is further supported by the observed reduction in activity levels among our participants, as evident by their pre-injury and current Tegner scores. Despite being on average at a stage of returning to sport (mean time post-surgery: 13.5 months), many participants had not resumed their previous activity level, a tendency often related to re-injury worries [10, 11, 41] .

To the authors’ knowledge, no previous study has provided a TSK-17 cutoff recommendation for individuals following ACL injury. Shortened versions of the TSK are frequently used instead of the original 17 items version. Particularly, in the TSK-11, items with low internal consistency have been removed, resulting in a concise version with comparable psychometric properties to the longer version, notably among patients with low back pain [42]. However, pilot work by Paterno et al. [14] did define a TSK-11 cutoff for ACL injured persons, with scores ≥ 17 interpreted as high kinesiophobia. This cutoff, equivalent to 26.5 on the TSK-17, is considerably lower than the established 38 for chronic pain [5]. Based on the ROC analysis, applying such a cutoff would have yielded 92.2% sensitivity and 36.6% specificity, resulting in many participants being incorrectly classified as having high re-injury worries despite low kinesiophobia. Moreover, it may be inadvisable to apply a TSK-11-based cutoff for TSK-17. Doing so would overlook six additional items that contribute to the TSK-17 score, combined with the limited internal consistency in the current sample. Moreover, the TSK-11 does not contain the fear-specific Q9 and thus lacks an explicit measure of fear of re-injury that does not refer to pain or general exercise as the cause of a potential future injury.

Kvist et al. [20] were the first to apply the TSK on individuals after ACL injury in 2005. However, the authors used an adapted version, in which multiple relevant modifications were made to better suit the ACL-injured athletic population [20]. The modified version, despite lacking formal evaluation of its psychometric properties, can be expected to better reflect re-injury worries in this population, given that the wording (e.g., “knee trouble” instead of “pain”) is more relatable. This version has been used in at least three other studies since then [17, 41, 43] and could potentially serve as a viable option for practitioners opting for continuity with the original instrument while maintaining its relevance for athletes. Kvist et al., 2013 [43] reported a moderate correlation between their modified TSK-17 and the ACL-RSI measure of psychological readiness, indicating that the two constructs are related to each other. In the present study, however, we found no significant correlations between the ACL-RSI and the original TSK version, indicating strong discriminant validity, confirming that the two measures address distinct constructs with limited overlap. The ACL-RSI, designed particularly for ACL-injured persons, contains items related to fear of re-injury, confidence and sport-specific risk appraisal, as well as references to worries regarding prolonged rehabilitation. While elements such as confidence and risk appraisal are reflected in the TSK as well, it is the context that is inherently different. Confidence in the TSK relates to general aversion of physical activity (e.g., “*I’m afraid that I injure myself if I exercise*” (Q1), “*No one should have to exercise when he/she is in pain*”) (Q17), which is less of a concern to athletes for whom physical activity may be a way of life [44]. Risk appraisal is deeply connected to pain and naturally feeds into catastrophizing thoughts due to statements such as: “*I wouldn’t have this much pain if there weren’t something potentially dangerous going on in my body*”. There are also statements concerning how “*people with a condition like mine*” should behave, which are absent from the ACL-RSI. Therefore, the lack of association between the measures, observed in the present study was not surprising.

It has been reported that athletes often struggle with frustration [45–47], anger [46], anxiety [45, 47] and depression [45–47] during the recovery process. These emotions are intensified by the realisation of how the injury has disrupted the athlete’s sports participation, along with the feeling of missing out on opportunities as their teammates continue to play [47]. Throughout the recovery process, pain (among other factors) may contribute to the athlete’s appraisal of the severity of their injury [48]. However, when the injury/surgery pain subsides, other factors affect their re-injury fear [17, 19], which can manifest particularly when required to perform risk-associated movements related to the injury context [47]. This is again fundamentally different from patients experiencing chronic pain, whose catastrophising thoughts may lead to avoidance behaviour [49]. The pain experience in those patients is central to the anxiety and behavioural change, which may manifest in avoiding movement in general, rather than in an injury-specific context [3]. Tissot et al., 2023 [50] demonstrated that the TSK (the 11-item version) underestimated task-specific fear even in individuals with low back pain. Questionnaires that target specific fears, regarding injury-specific situations, are seldom applied in research/clinical context. One example is an eight-item questionnaire proposed by Ardern et al., 2012 [51], which has shown high internal consistency and includes a question related to “*injury-provoking situations when playing your spor*t”, as well as referring to environmental conditions such as a wet playing field or the type of gym floor. Another injury anxiety assessment tool, the Japanese ACL-25 (JACL-25) questionnaire [52], contains task-specific items that refer to worries, fear and hesitation during e.g., hopping, changing direction, landing. Huang et al., 2019 [53] reported that the TSK only moderately correlated to the JACL-25, and was thus interpreted as having insufficient validity for individuals after ACL injury. Similarly, a lack of associations between the TSK to the ACL-RSI in the present study does not support administering the TSK to ACL-injured persons when there are other more relevant questionnaires.

Clinicians and researchers have at hand questionnaires which are more population-specific, such as the ACL-RSI 54, which has also a short 6-item version [54]. Although short, it captures the same concepts as the longer version and unlike the TSK, does not focus on pain or general exercise. Another tool when it comes to sports injuries, particularly among competitive athletes, is the re-injury anxiety inventory (RIAI) [55], which highlights the psychological state of an athlete in relation both to their rehabilitation and to future reintegration into competitive sports. However, the RIAI would not be suitable for recreational, non-competitive athletes as it has many items referring explicitly to return to competition. Clinicians may therefore benefit from selecting questionnaires that align with their patients’ needs, rather than defaulting to tools that are commonly used in studies. On that note, the authors would like to highlight the importance of delving into scientific methods when citing articles. Historically, this has been overlooked, resulting in a widespread use of the classic TSK on ACL-injured persons [9], instead of the modified version used by Kvist et al. [20].

### Study limitations

First, although the sex distribution was similar across subgroups, the overall sample included a higher proportion of females. Therefore, we cannot rule out possible gender-related differences in kinesiophobia reporting. Second, only a subset (*n* = 30) completed the ACL-RSI, which is below the recommended *n* = 50 [26], limiting the analysis of the TSK’s discriminant validity and its relationship with a more ACL-specific measure of psychological readiness across the entire cohort. A third limitation concerns the subgrouping, which was based on an arbitrary cutoff value of the KOOS_pain_ subscale. In the present study, a stringent 90% cutoff was used, which incidentally resulted in a balanced subgroup distribution. However, as this threshold was not empirically derived, we also repeated the analysis using the 86.1% cutoff reported in the literature [30], yielding comparable results *(supplementary material)* and thus supporting the robustness of our findings. Fourth, given that data from two separate projects were combined, differences in the order of how questionnaires were completed and their format (paper vs. digital) could potentially have introduced variability in their scores [56]. Finally, the use of convenience sampling may further limit the generalisability of the findings to other populations, e.g., older athletes.

## Conclusion and recommendations

The present findings indicate that the TSK may have limited suitability for assessing re-injury worries in active individuals after ACL reconstruction. The total score’s strong dependence on pain-specific items and limited internal consistency in pain-free individuals raise concerns about its use in this population. Furthermore, the TSK’s non-satisfying sensitivity compared with a more direct measure of re-injury worries and its divergence from the ACL-RSI highlight potential conceptual and psychometric limitations. From a research perspective, inclusion of the TSK in future data collections of ACL-injured persons should be carefully considered. Existing registries or datasets may also consider inspecting their TSK data alongside other measures of related constructs to explore, among other properties, the between-measurement relationships. Then, a more informed decision can be made regarding the interpretation of TSK scores for individuals after ACL injury. From a clinical perspective, clinicians and researchers may benefit from considering adopting more population-specific instruments when addressing re-injuries worries among ACL-injured persons, to better describe the psychological dimensions of this injury.

## Supplementary Information

Supplementary Material 1.

## Data Availability

Data will be available from the authors upon reasonable request and in accordance with GDPR regulations.

## References

[1] Kori SHM, Miller P, Todd DD. Kinesiophobia: a new view of chronic pain behavior. Pain Manage. 1990:35–43.

[2] Lethem J, Slade P, Troup J, Bentley G. Outline of a fear-avoidance model of exaggerated pain perception—I. Behav Res Ther. 1983;21(4):401–8. 6626110 10.1016/0005-7967(83)90009-8

[3] Vlaeyen JW, Linton SJ. Fear-avoidance and its consequences in chronic musculoskeletal pain: a state of the art. Pain. 2000;85(3):317–32. 10781906 10.1016/S0304-3959(99)00242-0

[4] Miller RP, Kori SH, Todd DD. The Tampa Scale: a measure of kinisophobia. Clin J Pain. 1991;7(1):51.

[5] Vlaeyen J, Kole-Snijders AM, Boeren RG, Van Eek H. Fear of movement/(re) injury in chronic low back pain and its relation to behavioral performance. Pain. 1995;62(3):363–72. 8657437 10.1016/0304-3959(94)00279-N

[6] Güney-Deniz H, Irem Kınıklı G, Çağlar Ö, Atilla B, Yüksel İ. Does kinesiophobia affect the early functional outcomes following total knee arthroplasty? Physiother Theory Pract. 2017;33(6):448–53. 28481125 10.1080/09593985.2017.1318988

[7] de Oliveira Silva D, Barton CJ, Briani RV, Taborda B, Ferreira AS, Pazzinatto MF, de Azevedo FM. Kinesiophobia, but not strength is associated with altered movement in women with patellofemoral pain. Gait Posture. 2019;68:1–5. 30408709 10.1016/j.gaitpost.2018.10.033

[8] Nijs J, Roussel N, Van Oosterwijck J, De Kooning M, Ickmans K, Struyf F, Meeus M, Lundberg M. Fear of movement and avoidance behaviour toward physical activity in chronic-fatigue syndrome and fibromyalgia: state of the art and implications for clinical practice. Clin Rheumatol. 2013;32(8):1121–9. 23639990 10.1007/s10067-013-2277-4

[9] Mir B, Vivekanantha P, Dhillon S, Cotnareanu O, Cohen D, de Nagai K. Fear of reinjury following primary anterior cruciate ligament reconstruction: a systematic review. Knee Surg Sports Traumatol Arthrosc. 2023;31(6):2299–314. 36562808 10.1007/s00167-022-07296-6

[10] Ardern CL, Webster KE, Taylor NF, Feller JA. Return to sport following anterior cruciate ligament reconstruction surgery: a systematic review and meta-analysis of the state of play. Br J Sports Med. 2011;45(7):596–606. 21398310 10.1136/bjsm.2010.076364

[11] Yensen K, Mayfield CK, Bolia IK, Palmer RA, Brown M, Kim DR, Abu-Zahra MS, Kotlier JL, Webb T, Cleary E. Subjective causes for failure to return to sport after anterior cruciate ligament reconstruction: a systematic review and meta-analysis. Sports Health. 2025:17(2):243-51. 10.1177/19417381241231631 PMC11569586 38532528

[12] Markström JL, Grinberg A, Häger CK. Fear of re-injury following anterior cruciate ligament reconstruction is manifested in muscle activation patterns of single-leg side-hop landings. Phys Ther. 2022:102.(2):pzab218. 10.1093/ptj/pzab218 PMC8860189 34554253

[13] Trigsted SM, Cook DB, Pickett KA, Cadmus-Bertram L, Dunn WR, Bell DR. Greater fear of reinjury is related to stiffened jump-landing biomechanics and muscle activation in women after ACL reconstruction. Knee Surg Sports Traumatol Arthrosc. 2018;26(12):3682–9. 29700560 10.1007/s00167-018-4950-2

[14] Paterno MV, Flynn K, Thomas S, Schmitt LC. Self-reported fear predicts functional performance and second ACL injury after ACL reconstruction and return to sport: a pilot study. J Sports health. 2018;10(3):228–33. 10.1177/1941738117745806 PMC5958451 29272209

[15] Tagesson S, Kvist J. Greater fear of re-injury and increased tibial translation in patients who later sustain an ACL graft rupture or a contralateral ACL rupture: a pilot study. J Sports Sci. 2016;34(2):125–32. 10.1080/02640414.2015.1035668 25894209

[16] Piussi R, Magnusson C, Andersson S, Mannerkorpi K, Thomeé R, Samuelsson K, Hamrin Senorski E. Some, but not all, patients experience full symptom resolution and a positive rehabilitation process after ACL reconstruction: an interview study. Knee Surg Sports Traumatol Arthrosc. 2023;31(7):2927–35. 10.1007/s00167-022-07271-1 PMC10276088 36484809

[17] Little C, Lavender AP, Starcevich C, Mesagno C, Mitchell T, Whiteley R, Bakhshayesh H, Beales D. Understanding fear after an anterior cruciate ligament injury: a qualitative thematic analysis using the common-sense model. Int J Environ Res Public Health. 2023;20(4):2920. 36833617 10.3390/ijerph20042920 PMC9957354

[18] Grinberg A. Reinjury Fear and Anxiety Following Anterior Cruciate Ligament Injury. Let’s Get Our Constructs Straight! JOSPT Open. 2023;1(2):1–4.

[19] Tjong VK, Murnaghan ML, Nyhof-Young JM, Ogilvie-Harris DJ. A qualitative investigation of the decision to return to sport after anterior cruciate ligament reconstruction: to play or not to play. Am J Sports Med. 2014;42(2):336–42. 24197615 10.1177/0363546513508762

[20] Kvist J, Ek A, Sporrstedt K. Good L. Fear of re-injury: a hindrance for returning to sports after anterior cruciate ligament reconstruction. Knee Surg Sports Traumatol, Arthrosc. 2005;13(5):393–7. 10.1007/s00167-004-0591-8 15703963

[21] George SZ, Lentz TA, Zeppieri G Jr, Lee D, Chmielewski TL. Analysis of shortened versions of the Tampa Scale for Kinesiophobia and Pain Catastrophizing Scale for patients following anterior cruciate ligament reconstruction. Clin J Pain. 2012;28(1):73. 21677565 10.1097/AJP.0b013e31822363f4 PMC3703641

[22] Karbalaie A, Strong A, Nordström T, Schelin L, Selling J, Grip H, Prorok K, Häger CK. Beyond self-reports after anterior cruciate ligament injury – machine learning methods for classifying and identifying movement patterns related to fear of re-injury. J Sports Sci. 2026;44(3):342–56. 10.1080/02640414.2025.2578584 41131712

[23] Webster KE, Feller JA, Lambros C. Development and preliminary validation of a scale to measure the psychological impact of returning to sport following anterior cruciate ligament reconstruction surgery. J Phys therapy sport. 2008;9(1):9–15. 10.1016/j.ptsp.2007.09.003 19083699

[24] Bonett DG. Sample size requirements for testing and estimating coefficient alpha. J educational Behav Stat. 2002;27(4):335–40.

[25] French DJ, France CR, Vigneau F, French JA, Evans RT. Fear of movement/(re) injury in chronic pain: a psychometric assessment of the original English version of the Tampa scale for kinesiophobia (TSK). Pain. 2007;127(1–2):42–51. 16962238 10.1016/j.pain.2006.07.016

[26] Terwee CB, Bot SD, de Boer MR, Van der Windt DA, Knol DL, Dekker J, Bouter LM, de Vet HC. Quality criteria were proposed for measurement properties of health status questionnaires. J Clin Epidemiol. 2007;60(1):34–42. 17161752 10.1016/j.jclinepi.2006.03.012

[27] The Swedish knee. ligament registry - Annual report 2020 [www.aclregister.nu]. https://www.aclregister.nu/media/uploads/Annual%20reports/rapport2020en.pdf.

[28] Roos EM, Roos HP, Lohmander LS, Ekdahl C, Beynnon BD. Knee Injury and Osteoarthritis Outcome Score (KOOS)--development of a self-administered outcome measure. J Orthop Sports Phys Ther. 1998;28(2):88–96. 9699158 10.2519/jospt.1998.28.2.88

[29] van Meer BL, Meuffels DE, Vissers MM, Bierma-Zeinstra SM, Verhaar JA, Terwee CB, Reijman M. Knee injury and Osteoarthritis Outcome Score or International Knee Documentation Committee Subjective Knee Form: which questionnaire is most useful to monitor patients with an anterior cruciate ligament rupture in the short term? Arthroscopy: J Arthroscopic Relat Surg. 2013;29(4):701–15. 10.1016/j.arthro.2012.12.015 23402944

[30] Lohmander L, Östenberg A, Englund M, Roos H. High prevalence of knee osteoarthritis, pain, and functional limitations in female soccer players twelve years after anterior cruciate ligament injury. Arthritis Rheumatism: Official J Am Coll Rheumatol. 2004;50(10):3145–52. 10.1002/art.20589 15476248

[31] Tegner Y. J <>Lysholm 1985 Rating systems in the evaluation of knee ligament injuries. Clin Orthop Relat Res 198 43–9. 4028566

[32] Harris PA, Taylor R, Thielke R, Payne J, Gonzalez N, Conde JG. Research electronic data capture (REDCap)—a metadata-driven methodology and workflow process for providing translational research informatics support. J Biomed Inform. 2009;42(2):377–81. 18929686 10.1016/j.jbi.2008.08.010 PMC2700030

[33] Mukaka MM. A guide to appropriate use of correlation coefficient in medical research. Malawi Med J. 2012;24(3):69. 23638278 PMC3576830

[34] Bellamy N. Principles of clinical outcome assessment. 2014.

[35] Cohen J. Statistical power analysis for the behavioral sciences. 2 ed. Routledge; 1988. pp. 20–7.

[36] Landis, JR, Koch, GG. The measurement of observer agreement for categorical data. Biometrics. 1977;159–74. 843571

[37] Çorbacıoğlu ŞK, Aksel G. Receiver operating characteristic curve analysis in diagnostic accuracy studies: A guide to interpreting the area under the curve value. Turkish J Emerg Med. 2023;23(4):195–8. 10.4103/tjem.tjem_182_23 PMC10664195 38024184

[38] Tavakol M, Dennick R. Making sense of Cronbach’s alpha. Int J Med Educ. 2011;2:53. 28029643 10.5116/ijme.4dfb.8dfd PMC4205511

[39] Lundberg M, Styf J, Jansson B. On what patients does the Tampa Scale for Kinesiophobia fit? Physiother Theory Pract. 2009;25(7):495–506. 19925172 10.3109/09593980802662160

[40] Luque-Suarez A, Martinez-Calderon J, Falla D. Role of kinesiophobia on pain, disability and quality of life in people suffering from chronic musculoskeletal pain: a systematic review. Br J Sports Med. 2019;53(9):554–9. 29666064 10.1136/bjsports-2017-098673

[41] Ardern CL, Österberg A, Tagesson S, Gauffin H, Webster KE, Kvist J. The impact of psychological readiness to return to sport and recreational activities after anterior cruciate ligament reconstruction. J Br J Sports Med. 2014;48(22):1613–9. 25293342 10.1136/bjsports-2014-093842

[42] Woby SR, Roach NK, Urmston M, Watson PJ. Psychometric properties of the TSK-11: a shortened version of the Tampa Scale for Kinesiophobia. Pain. 2005;117(1–2):137–44. 16055269 10.1016/j.pain.2005.05.029

[43] Kvist J, Österberg A, Gauffin H, Tagesson S, Webster K, Ardern C. Translation and measurement properties of the S wedish version of ACL-Return to Sports after Injury questionnaire. Scand J Med Sci Sports. 2013;23(5):568–75. 22257241 10.1111/j.1600-0838.2011.01438.x

[44] Brewer BW. Handbook of sports medicine and science: sport psychology. Wiley. 2009;(12):113–20.

[45] Leddy MH, Lambert MJ, Ogles BM. Psychological consequences of athletic injury among high-level competitors. Res Q Exerc Sport. 1994;65(4):347–54. 7886284 10.1080/02701367.1994.10607639

[46] Smith AM, Scott SG, O’FALLON WM, Young ML. Emotional responses of athletes to injury. Mayo Clin Proc. 1990;65(1):38–50. 2296211 10.1016/s0025-6196(12)62108-9

[47] Johnston LH, Carroll D. The context of emotional responses to athletic injury: a qualitative analysis. J Sport Rehabilitation. 1998;7(3):206–20.

[48] Blackburn T, Pietrosimone B, Goodwin JS, Johnston C, Spang JT. Co-activation during gait following anterior cruciate ligament reconstruction. Clin Biomech Elsevier Ltd. 2019;67:153–9. 10.1016/j.clinbiomech.2019.05.010 31121428

[49] Zusman M. Mechanisms of peripheral neuropathic pain: implications for musculoskeletal physiotherapy. Phys Therapy Reviews. 2008;13(5):313–23.

[50] Tissot L-PM, Evans DW, Kirby E, Liew BXW. Tampa Scale of Kinesiophobia may underestimate task-specific fear of movement in people with and without low back pain. Pain Rep. 2023;8(4):e1081. 37293339 10.1097/PR9.0000000000001081 PMC10247215

[51] Ardern CL, Taylor NF, Feller JA. Webster KE. Fear of re-injury in people who have returned to sport following anterior cruciate ligament reconstruction surgery. 2012;15(6):488–95. 10.1016/j.jsams.2012.03.015 22695136

[52] Nagao M, Doi T, Saita Y, Kobayashi Y, Kubota M, Kaneko H, Takazawa Y, Ishijima M, Kurosawa H, Kaneko K. A novel patient-reported outcome measure for anterior cruciate ligament injury: evaluating the reliability, validity, and responsiveness of Japanese anterior cruciate ligament questionnaire 25. Knee Surg Sports Traumatol Arthrosc. 2016;24:2973–82. 25894748 10.1007/s00167-015-3595-7

[53] Huang H, Nagao M, Arita H, Shiozawa J, Nishio H, Kobayashi Y, Kaneko H, Nagayama M, Saita Y, Ishijima M. Reproducibility, responsiveness and validation of the Tampa Scale for Kinesiophobia in patients with ACL injuries. Health Qual Life Outcomes. 2019;17(1):1–8. 31506078 10.1186/s12955-019-1217-7 PMC6737637

[54] Webster KE, Feller JA. Development and validation of a short version of the Anterior Cruciate Ligament Return to Sport After Injury (ACL-RSI) scale. J Orthop J sports Med. 2018;6(4):2325967118763763. 29662909 10.1177/2325967118763763 PMC5894922

[55] Walker N, Thatcher J, Lavallee D. A preliminary development of the Re-Injury Anxiety Inventory (RIAI). Phys Ther Sport. 2010;11(1):23–9. 20129120 10.1016/j.ptsp.2009.09.003

[56] Koho P, Aho S, Kautiainen H, Pohjolainen T, Hurri H. Test–retest reliability and comparability of paper and computer questionnaires for the Finnish version of the Tampa Scale of Kinesiophobia. Physiotherapy. 2014;100(4):356–62. 24679373 10.1016/j.physio.2013.11.007

