# Supplementary material for: Rethinking the Tampa scale of kinesiophobia as a measure of re-injury worries after anterior cruciate ligament injury
Source: BMC Sports Sci Med Rehabil. 2026 Apr 6;18:196. doi: 10.1186/s13102-026-01684-y (PMC13081327; doi:10.1186/s13102-026-01684-y)
Supplement: Supplementary file 1 — Supplementary Material 1. [file 13102_2026_1684_MOESM1_ESM.docx]

**Supplementary analysis – Determining the best cutoff of the TSK-17 against TSK_Q9_ classification**

With no current gold-standard measure for fear of re-injury, the goal of this exploratory analysis was to determine the classification performance of the TSK_total_ in individuals with ACL injury, by testing multiple cutoffs against classification based on TSK_Q9_ – *“I am afraid that I might injure myself accidentally”* – according to which, scores of 1-2 are considered “low-fear” and 3-4 are considered high-fear [1].

For this purpose, we performed a receiver operating curve (ROC) analysis. The optimal cutoff was determined as the point on the ROC curve with the best sensitivity and specificity based on the maximum Youden J statistic. The ROC curves for the TSK_total_ are visualised in Figure 4b in the main text.

Table S-1 details all TSK_total_ cutoffs and their ROC coordinates. TSK_total_ Cutoff with the maximal Youden’s index is highlighted.

**Table S-1.**

| **Coordinates of the ROC Curve** | | | |
| --- | --- | --- | --- |
| Test Result Variable(s): TSK_total_ | | | |
| Positive if Greater Than or Equal To^a^ | Sensitivity | 1 - Specificity | Youden's Index |
| 16.0000 | 1.000 | 1.000 | .000 |
| 18.5000 | 1.000 | .976 | .024 |
| 20.5000 | 1.000 | .927 | .073 |
| 21.5000 | .980 | .854 | .127 |
| 22.5000 | .980 | .829 | .151 |
| 23.5000 | .980 | .805 | .176 |
| 24.5000 | .941 | .805 | .136 |
| 25.5000 | .922 | .732 | .190 |
| 26.5000 | .922 | .634 | .287 |
| 27.5000 | .902 | .610 | .292 |
| 28.5000 | .863 | .561 | .302 |
| 29.5000 | .843 | .463 | .380 |
| 30.5000 | .745 | .415 | .330 |
| 31.5000 | .725 | .293 | .433 |
| 32.5000 | .725 | .268 | .457 |
| 33.5000 | .706 | .122 | .584 |
| 34.5000 | .569 | .098 | .471 |
| 35.5000 | .490 | .098 | .393 |
| 36.5000 | .431 | .073 | .358 |
| 37.5000 | .373 | .049 | .324 |
| 38.5000 | .333 | .049 | .285 |
| 39.5000 | .255 | .000 | .255 |
| 40.5000 | .196 | .000 | .196 |
| 41.5000 | .176 | .000 | .176 |
| 42.5000 | .118 | .000 | .118 |
| 43.5000 | .098 | .000 | .098 |
| 44.5000 | .059 | .000 | .059 |
| 47.5000 | .039 | .000 | .039 |
| 51.0000 | .020 | .000 | .020 |
| 53.0000 | .000 | .000 | .000 |
| The test result variable(s): TSK_total_ has at least one tie between the positive actual state group and the negative actual state group. | | | |
| a. The smallest cutoff value is the minimum observed test value minus 1, and the largest cutoff value is the maximum observed test value plus 1. All the other cutoff values are the averages of two consecutive ordered observed test values. | | | |

**Supplementary analysis – Internal consistency of the TSK-17 for 86.1% cutoff of KOOS_pain_ [2]**

To confirm that the findings were not dependent on this threshold, we also performed a supplementary analysis using Cronbach’s α on subgroups determined based on the previously published 86.1% cutoff.

NO-PAIN (n = 56): α = 0.643 (non-satisfactory internal consistency).

PAIN (n = 36): α = 0.780 (satisfactory internal consistency).

1. Markström JL, Grinberg A, Häger CK: **Fear of re-injury following anterior cruciate ligament reconstruction is manifested in muscle activation patterns of single-leg side-hop landings**. *Phys Ther* 2022.

2. Lohmander L, Östenberg A, Englund M, Roos H: **High prevalence of knee osteoarthritis, pain, and functional limitations in female soccer players twelve years after anterior cruciate ligament injury**. *Arthritis & Rheumatism: Official Journal of the American College of Rheumatology* 2004, **50**(10):3145-3152.
